# Pretreatment by omalizumab allows allergen-specific immunotherapy in children and young adult with severe allergic asthma

**DOI:** 10.1186/2045-7022-5-S2-P1

**Published:** 2015-03-23

**Authors:** Nathalie Lambert, Tamazoust Guiddir, Jocelyne Just, Flore Amat

**Affiliations:** 1 Armand Trousseau Children's Hospital, Allergology Department, Paris, France

## Background

Subcutaneous allergen-specific immunotherapy (SCIT) is a valuable treatment option for patients with controlled mild to moderate allergic asthma However, SCIT is contraindicated for patients with severe persistent asthma due to a potential systemic allergic reaction. Several studies in adolescents and adults with persistent allergic rhinitis and moderate persistent allergic asthma have shown that SCIT is better tolerated when combined with. Nevertheless, no previous studies have been conducted in children and adolescents with severe asthma to assess the safety and efficacy of a combination treatment of SCIT and omalizumab.

## Methods

We report here the observations of six patients, aged 11 to 21 years, with severe persistent asthma controlled by omalizumab as add-on therapy who received SCIT under a cluster protocol during omalizumab treatment and then SCIT maintenance alone after discontinuation of omalizumab.

## Results

Although no patients experienced severe exacerbation, one patient had to stop SCIT after one month of treatment because of uncontrolled asthma. For the five remaining patients, asthma control continued to improve during the combined treatment with SCIT and omalizumab (median time duration = 8 months) despite a decrease in maintenance treatment for all of them. SCIT was continued alone for a median time of 25.5 months and was well tolerated. For these patients, asthma was totally controlled and therapeutic levels of maintenance treatment could be further reduced

## Conclusion

Pretreatment by omalizumab for patients with persistent severe allergic asthma seems to improve the safety and probably also the efficacy of SCIT. These results open up perspectives of SCIT for children suffering from severe allergic asthma. However,the primary outcomes of these observations are not included in a standardized protocol and will require further confirmation by prospective double blind studies.

